# LVI and DI-SPME Combined with GC/MS and GC/MS for Volatile Chemical Profile Investigation and Cytotoxic Power Evaluation of Essential Oil and Hydrolate from *Cannabis sativa* L. cv. Carmagnola

**DOI:** 10.3390/molecules29143299

**Published:** 2024-07-12

**Authors:** Vittorio Vinciguerra, Marta Di Martile, Monica Mollica Graziano, Donatella Del Bufalo, Stefania Garzoli

**Affiliations:** 1 Department for Innovation in Biological Systems, Food and Forestry, University of Tuscia, 01100 Viterbo, Italy; vincigue@unitus.it (V.V.); monicamollica70@gmail.com (M.M.G.); 2 Preclinical Models and New Therapeutic Agents Unit, IRCCS Regina Elena National Cancer Institute, 00144 Rome, Italy; marta.dimartile@ifo.it (M.D.M.); donatella.delbufalo@ifo.it (D.D.B.); 3 Department of Drug Chemistry and Technology, Sapienza University, 00185 Rome, Italy

**Keywords:** chemical analysis, LVI-GC/MS, DI-SPME-GC/MS, cytotoxicity

## Abstract

*Cannabis sativa* L. is a plant that has been cultivated since ancient times thanks to its various uses. Even its extraction products, such as essential oil and hydrolate, having a varied chemical composition and rich in bioactive components, find wide use in different sectors, gathering ever-increasing interest over time. In this work, the essential oil of *Cannabis sativa* L. cv. Carmagnola was characterized by using Gas Chromatography/Mass Spectrometry (GC/MS) and, for the first time, the chemical profile of the hydrolate was also described through different analytical techniques such as Large-Volume Injection Gas Chromatography/Mass Spectrometry (LVI-GC/MS) and Direct Immersion-Solid Phase Microextraction-Gas Chromatography/Mass spectrometry (DI-SPME-GC/MS), in order to provide a more complete compositional profile. The results of the analyses conducted on the hydrolate highlighted a high content of α-terpineol; on the other side, in the essential oil, a prevalence of monoterpenes, with α-pinene and limonene as the characterizing components, was detected. Both matrices were also investigated to evaluate their cytotoxic activity by using a panel of cancer cell lines derived from different histotypes such as melanoma (A375, LOX IMVI), non-small cell lung cancer (H1299, A549), colon (HT29) and pancreatic (L3.6) cancer cell lines. The obtained data demonstrated that essential oil was more effective than hydrolate in terms of reduction in cell viability.

## 1. Introduction

*Cannabis sativa* L. (Cannabis or hemp) is an annual herbaceous plant of the Cannabaceae family [1]. Initially it was only a dioecious species, i.e., with female and male flowers on different individuals, but over time monoecious genotypes also developed, which bear male and female flowers on the same individual [2]. The point of origin of cannabis is believed to be in Asia [3]. The multiple uses of the plant, particularly as a source of fiber, as well as its medicinal and narcotic uses, have led to an immense use of the plant throughout the world [4]. Its secondary metabolites such as terpenoids, flavonoids, sterols and phytocannabinoids are responsible for the various effects of cannabis.

In recent years, hemp essential oil (EO) extraction has gained increasing interest due to its various fields of application. In fact, it has been widely used as an insecticide [5] and as a control agent against invasive weed germination thanks to its allelopathic effects [6]. In recent years it has also been used in the food sector as a flavoring agent for drinks [7].

Cannabis EO is a complex mixture of volatile compounds characterized by monoterpenes, terpenoids, sesquiterpenes and diterpenes [8,9,10]. Each single component is responsible for a characteristic fragrance so as to determine a specific aromatic profile for each variety. Many factors, such as environmental and climatic conditions, genotype, cultivation technique, harvesting and storage conditions can influence the composition of EO [11,12,13,14]. All this means that essential oils (EOs) extracted even from the same variety can have a partially comparable and not entirely overlapping chemical profile.

Many of the main terpene compounds of hemp EO have been reported to induce antidepressant, relaxing, anxiolytic, sedative but also antimicrobial, antioxidant and anticancer effects [15,16].

Hydrolate (Hy), known as hydrosol or aromatic water, is a distillate obtained after the distillation process of essential oil [17]. The hydrolates (Hys) consist of a predominantly aqueous phase and an oily phase, i.e., an emulsion of EO droplets, and are easy to obtain as they are produced in high quantities during the distillation phase and are generally considered milder in their properties than the corresponding EOs because they are more diluted. Initially, they were only considered a by-product or even a waste but, over time, the interest in Hys, both for their composition and for their biological properties, has grown exponentially, also because much of the information about it is still fragmented. However, over time, they have been shown to possess various biological properties such as antibacterial effects. Furthermore, their composition makes them easily exploitable in the food, cosmetic and pharmaceutical sectors [18,19,20].

Taking into account the ever-increasing demand for natural cannabis-based products for medical uses, it is essential to increase knowledge on the chemical aspects and beneficial effects, such as the anti-cancer effect, of hemp extraction products. In the literature, there are few studies that report the description of the compositional profile of *C. sativa* Hys and none concerning the chemical characterization of the Hy from the Carmagnola variety. Given this, in our study, with the aim of providing detailed information on the qualitative and semi-quantitative composition of the Hy of *C. sativa* Carmagnola cv. grown in Italy, we used two different analytical methodologies, namely, Large-Volume Injection Gas Chromatography/Mass Spectrometry (LVI-GC/MS, Sant Cugat del Vallés, Barcelona, Spain) and Direct Immersion-Solid Phase Microextraction Gas Chromatography/Mass Spectrometry (DI-SPME-GC/MS, Perkin Elmer, CT, USA). On the other side, the chemical composition of EO was obtained by direct injection in a GC-MS apparatus. Furthermore, the cytotoxic effect of both EO and Hy has been evaluated in a panel of tumor cell lines from different origins and in the fibroblast cell line BJ-hTERT.

The findings of this study can be the starting point for developing future research in order to broaden knowledge on the compositional profile of cannabis and to optimize extraction methods in relation to the compounds mainly characterizing its extraction products such as EO and Hy.

## 2. Results and Discussion

### 2.1. Chemical Composition of C. sativa EO

By GC/MS chemical analyses, the chemical profile of *C. sativa* EO was described (Table 1). The monoterpene fraction (82.0%) was prevalent over the sesquiterpene fraction (17.5%), with *α*-pinene (35.8%) and limonene as main constituents (27.6%). *β*-Caryophyllene reached 9.6% and represented the most abundant sesquiterpene followed by humulene (1.9%) and *β*-bisabolene (1.3%) while all the others ranged from 0.1% to 0.8%.

As reported in previous works, some of the most abundant terpene compounds present in cannabis EO are myrcene, *β*-caryophyllene, *α*-pinene and *α*-humulene [21]. In particular, in our work, the obtained data highlighted a high quantity of limonene. This monoterpene is also known to have anti-inflammatory effects [22]. Previous studies reported the presence of both limonene (5.45%; 0.7%) and *α*-pinene (13.45%; 2.1%), but with lower percentage values [23,24]. These quantitative variations in the composition of the essential oil may be due to different parameters such as genetics, cultivation practice, harvesting, storage, the initial physical state of the material and the extraction method applied to obtain the EO [11,13].

### 2.2. LVI-GC/MS Chemical Composition of C. sativa L. Hy

By LVI-GC/MS analyses, twelve components were detected and identified in Hy (Table 2). Among these, *α*-terpineol (14.5%) was the main detected monoterpene followed by linalool (3.2%) and fenchol (1.2%). The relatively more abundant compound was 2-methylisoborneol (19.2%), a non-canonical monoterpene, known to be responsible, together with geosmin, for the musty and earthy odor of water caused by various microorganisms [25]. This compound was not present in the EO, as well as other compounds such as limonene-1,2-diol (5.2%), 8-hydroxylinalool (4.2%), sobrerol acetate (9.6%) and sobrerol (6.4%). These latter two had already been detected in the Hy of the resin of *Pistacia lentiscus* [26]. In particular, sobrerol is a monoterpene with two hydroxyl functions known for its radical scavenging activity [27]. Limonene-1,2-diol is a monoterpenoid to which anti-inflammatory and antifungal activities have been attributed [28,29]. This bioactive compound, in addition to being naturally present in some plants [30], can also be produced by the biotransformation of limonene [30]. 8-Hydroxylinalool is a linalool-derived compound widely present in *Vitis vinifera* [31] and, as recently reported, in *Camellia sinensis* var. *assamica* ‘Hainan dayezhong’ [32]. This terpene oxide was demonstrated to have a special anti-microbial activity against Gram (±) bacteria strains and against pathogenic fungi [33].

No sesquiterpenes were detected. Other non-terpene components were identified with relevant average percentage values such as 4-OH-butanoic acid (10.0%) and 3-buten-1,2-diol-1(2-furanyl)-2-methyl (16.1%).

The presence of both low-polarity volatile compounds and low-volatility hydrophilic compounds makes complex the analysis of the Hy.

Most studies apply a liquid–liquid extraction with an organic solvent and subsequent introduction into a GC system. But, to the best of our knowledge, only one paper has reported the use of direct hydrosol analysis by GC-MS with encouraging results [34]. In our case, the use of a TOTAD (Through Oven Transfer Adsorption Desorption) interface allows high quantities (up to a few hundred microliters) of aqueous sample to be injected directly into a capillary gas chromatography column, overcoming the limitations of the injectable volume and providing the possibility of significantly increasing sensitivity if the Hys are very diluted.

### 2.3. DI-SPME/GC-MS Chemical Composition of C. sativa L. Hy

The sampling and analysis method applied to *C. sativa* Hy allowed the identification of twenty-two volatile components (Table 3). The monoterpene fraction (88.6%) was significantly higher than the sesquiterpene fraction (7.8%). According to the data obtained by LVI-GC/MS, *α*-terpineol (26.4%) was the principal monoterpene. It has countless biological activities including an anticancer effect [35]. Following this, linalool (23.2%) and fenchol (21.2%) were the compounds detected with a similar relative abundance. Among these oxygenated monoterpenes, it has been recently reported that linalool induces apoptosis of cancer cells via oxidative stress, and at the same time protects normal cells [36] while fenchol is known to possess antibacterial activity at a broad spectrum [37]. On the other hand, among the sesquiterpenes, *β*-caryophyllene, although with relatively low percentage values, was the most abundant. From the obtained data, it can be stated that DI-SPME/GC-MS compared to LVI/GC-MS, was the most adequate technique for the detection of the sesquiterpene component. On the other side, the LVI/GC-MS technique allowed the detection of other monoterpene compounds such as limonene-1,2-diol, 8-hydroxylinalool and sobrerol.

To the best of our knowledge, the present work is the first to have investigated the compositional profile of the Hy of *C. sativa* Carmagnola cv. In fact, in the scientific literature, one paper is available that describes the chemical composition of *C. sativa* Hy, but concerning the Kompolti variety [38].

### 2.4. EO and Hy Differently Affected the Viability of Tumor Cell Lines

**Figure 1:**
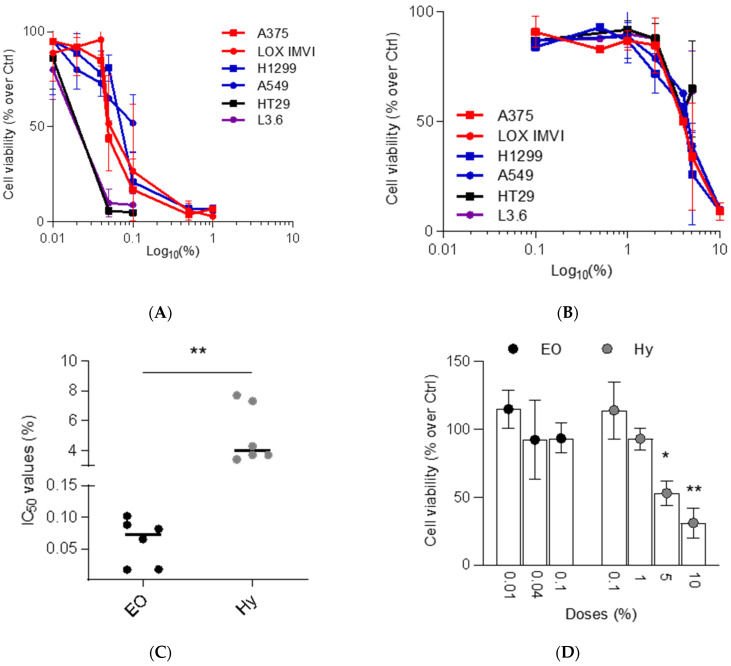
(**A**,**B**) Analysis of cell viability by MTT assay of a panel of tumor cell lines (A375, LOX IMVI, H1299, A549, HT29, L3.6) treated with the indicated concentrations of (**A**) EO or (**B**) Hy for 72 h. (**C**) IC_50_ values of tumor cells treated as reported in (**A**,**B**). (**D**) Analysis of cell viability by MTT assay of BjhTERT cells treated for 72 h with the indicated concentrations of EO or Hy. (**A**–**D**) The results are reported as “viability of treated cells/viability of control cells (Ctrl)” × 100, as the mean ± SD of three independent experiments. *p*-values were calculated between control and treated cells. (**C**,**D**) * *p* < 0.05, ** *p* < 0.01.

To evaluate the cytotoxic effect of EO and Hy, we tested their anticancer efficacy by using a panel of tumor cell lines derived from different histotypes. In particular, melanoma (A375, LOX IMVI), non-small cell lung cancer (H1299, A549), colon (HT29) and pancreatic (L3.6) cancer cell lines were exposed to increasing concentrations of EO (from 0.01 to 1%) or Hy (from 0.1 to 10%) for 72 h (Figure 1A–C, Table 1). EO and Hy induced a strong reduction in cell viability, with the IC_50_ of EO ranging from 0.017 to 0.101% (Figure 1A,C, Table 1) and that of Hy ranging from 3.430 to 7.702% (Figure 1B,C, Table 4). Colon and pancreatic cancer cells were more sensitive to EO treatment than the others, while all cell lines seemed to display a more homogeneous response when treated with Hy. Unexpectedly, colon and pancreatic cancer cells were the most sensitive to EO and the most resistant to Hy. As expected, considering the composition of EO and Hy, tumor cell lines were significantly more sensitive to EO than to Hy (Figure 1C). In fact, caryophyllene, humulene, α-pinene and linalool, found in EO with significantly higher percentage values than in the Hy, have been reported to possess anticancer activity [39]. Further, limonene, detected in EO as the second most abundant compound and missing in Hy, is known to have low toxicity and some inhibitory activity on the growth of tumor cells, as it is able to act on both the initiation and promotion phases [40]. Limonene has been defined as a natural molecule with pleiotropic pharmacological activity as it acts on various signaling pathways [41] and on different types of tumors [42]. Over time, the ever-increasing use of EOs has led to an increase in reports of allergic reactions [43]. Among the most abundant components, limonene and α-pinene are the components that could probably induce contact dermatitis (ACD) while β-caryophyllene could induce allergic reactions if inhaled [44,45]. Should a clinical trial evaluate the efficacy of EO or Hy from *C. sativa* be initiated as complementary or supportive therapy in the treatment of cancer, patients with known allergies to EO would be excluded from the study. Furthermore, patients would be informed about the possibility of developing allergic reactions and would be invited to pay attention to the possible appearance of symptoms and, therefore, to discontinue treatment.

Next, to assess whether the cytotoxic effect reported for EO and Hy was specific to tumor cells, we exposed BJhTERT, an immortalized normal fibroblast cell line, to the same concentrations of EO or Hy that we used for tumor cells for 72 h. Importantly, EO until 0.1% did not significantly reduce the BJhTERT cell viability (Figure 1D). On the contrary, Hy significantly reduced the fibroblast viability starting from 5% (Figure 1D), indicating that Hy similarly affected the viability of normal and tumor cells. However, considering the pool of bioactive compounds found, for the first, time, in *C. sativa* Hy, further experiments are needed to better explore this evidence and to investigate whether Hy induces off-target effects in other normal cells.

## 3. Materials and Methods

### 3.1. Plant Material

*C. sativa* essential oil and hydrolate from the Carmagnola cultivar were a kind gift of Aeroponica Perrotta Farm—Nola (NA) 80035, Italy.

### 3.2. LVI-GC-MS Analysis of C. sativa Hydrolate

Direct injection in a GC-MS system of large aqueous volume was performed using a Konik instrumentation equipped with a 5000C gas chromatograph coupled to a mass spectrometer detector MS Q2 (Sant Cugat del Vallés, Barcelona, Spain) and equipped with a TOTAD (Through Oven Transfer Adsorption Desorption) interface, operating as described in [46] with minor modifications. The adsorption temperature was kept at 60 °C for 6 min under a nitrogen gas flow of 150 mL/min to eliminate the solvent (water) present in the Hy sample (20 µL), which was transferred to the interface with a methanol flow of 0.1 mL/min for 1 min. The analytes were desorbed at 260 °C for 5 min.

The gas chromatograph operated with a Macherey-Nagel (Düren, Germany) fused-silica (30 m × 0.25 mm i.d.) column coated with 14% cyanopropylphenyl, 86% dimethylpolysiloxane (1701, film thickness 0.25 µm) and with a carrier gas helium at 2.0 mL/min constant flow. During the adsorption phase the oven was kept at 38 °C, then increased up to 245 °C at a rate of 10 °C/min.

The mass spectrometry interface was kept at 260 °C, the ion source operated at 180 °C in electron impact mode at 70 eV and MS analyzer in full scan mode from 41 to 400 *m*/*z*.

The compounds’ identification was performed by mass spectra interpretation, by comparison with the NIST20 computer library and the literature data [47]. Semiquantitative analysis (% relative concentration) was performed using the same Konik instrumentation and the same GC and TOTAD conditions. The mass spectrometric detector was replaced with a flame ionization detector (FID) operating at 280 °C. The analyses were carried out in triplicate.

### 3.3. DI-SPME/GC-MS Analysis of C. sativa Hy

To describe the chemical profile of the headspace from the Hy, direct immersion solid-phase microextraction (DI-SMPE) sampling followed by GC-MS technique was performed. About 2.0 mL of Hy was placed into a 7 mL glass vial with a PTFE-coated silicone septum. In order to reach thermal equilibrium, a thermostatic bath for 15 min with constant magnetic stirring was utilized. For the extraction of compounds, a SPME device from Supelco (Bellefonte, PA, USA) with 1 cm fiber coated with 50/30 μm DVB/CAR/PDMS (divinylbenzene/carboxen/polydimethylsiloxane) was chosen [48]. Before use, the fiber was conditioned at 270 °C for 30 min. After conditioning, it was immersed directly in the aqueous solution for 20 min at 60 °C for the extraction of volatile and semi-volatile compounds. For the extraction of the adsorbed compounds, the fiber was then inserted into the injector of the GC maintained at 250 °C. A Clarus 500 model Perkin Elmer (Waltham, MA, USA) gas chromatograph coupled with a mass spectrometer and equipped with an FID (flame detector ionization) was used for the analyses. For the separation of compounds, a Varian Factor Four VF-1 (60 m × 0.32 mm ID, DF = 1.0 μm) capillary column was housed in the GC oven. The chromatographic conditions were as follows: 50 °C up to 220 °C for 10 min at a rate of 6 °C min^−1^. A gas carrier was used with the flow rate of 1.0 mL min^−1^. The mass spectra were obtained in the electron impact mode (EI) at 70 eV in scan mode ranging 35–400 *m*/*z*.

The volatile compounds were identified by the comparison of the MS-fragmentation pattern of the analytes with those of pure components stored in the Wiley 2.2 mass spectra library database. Further, the linear retention indices (LRIs) were calculated using a series of alkane standards and compared with those reported in the literature. The relative amounts of the components were expressed as percent peak area relative to total peak area without the use of an internal standard and any factor correction. The analyses were carried out in triplicate.

### 3.4. GC-MS Analysis of C. sativa EO

The chemical characterization of *C. sativa* EO was obtained by GC/MS analysis using the same apparatus described above. In this case, the temperature program was set as follows: 60 °C up to 220 °C for 20 min at a rate of 6 °C min^−1^. The identification and quantification of the components were performed as reported in the previous Section 2.3. The analyses were conducted in triplicate.

### 3.5. Cell Cultures

Human melanoma (A375, LOX IMVI), non-small cell lung (H1299, A549), colon (HT29) and pancreatic (L3.6) cancer cell lines were cultured in RPMI-1640 medium (Euroclone, Milan, Italy) supplemented with 10% inactivated fetal bovine serum (FBS) (Gibco, Thermo Fisher Scientific, Waltham, MA, USA), 100 μg/mL penicillin/streptomycin (Euroclone) and 1% L-glutamine (Euroclone). Human telomerase reverse transcriptase immortalized fibroblasts (BJ-hTERT) were cultured in Dulbecco’s Modified Eagle’s medium supplemented with 10% inactivated fetal bovine serum, 100 μg/mL penicillin/streptomycin and 1% L-glutamine. Cells had been recently tested for mycoplasma contamination and authenticated through the analysis of STR profile.

### 3.6. Treatments and Analysis of Cell Viability

3 × 10^3^ cell were plated in 96 well plates and, after 24 h, were treated with increasing concentrations of EO or Hy. The latter were serially diluted in complete medium. Cell viability was evaluated by measuring 3-[4,5-dimethylthiazol-2-yl]-2,5-diphenyltetrazolium bromide inner salt (MTT, Sigma-Aldrich, St. Louis, MO, USA) dye absorbance after 72 h of treatment. Experiments were repeated at least three times with six technical replicates ran on the same plate.

### 3.7. Statistical Analysis

Statistical analyses and IC_50_ calculations were performed with GraphPad Prism 6.1 software. The experiments were replicated at least three times, unless otherwise indicated, and the data were expressed as average ± standard deviation (SD). Differences between groups, analyzed with T-test, 2-way ANOVA or Mann–Whitney, were considered statistically significant for *p* < 0.05.

## 4. Conclusions

Essential oils and hydrolates are a complex mixture of volatile compounds thanks to which they can be considered high-value derivative by-products. In our study, in order to obtain detailed compositional information on the Carmagnola cultivar, different and innovative methodological approaches have been carried out. The findings highlighted a profile qualitatively and quantitatively richer in terpene compounds in the EO than in the Hy never investigated until now. In line with this observation, the performed assays to evaluate the cytotoxic effects demonstrated that EO was more effective than Hy in the ability to reduce cancer cell viability. Further experiments aimed to expand the panel of tumor cells lines are needed to confirm these observations.

However, this preliminary study provides information on the compositional profile and bioactive potential usefulness for a more conscious use of these matrices and especially of *C. sativa* Hy, considered primarily as a by-product of the distillation process and therefore little exploited.

## Figures and Tables

**Table 1:** Chemical composition (percentage mean value ± standard deviation) of *C. sativa* L. EO.

N°	COMPONENT ^1^	LRI ^2^	LRI ^3^	EO
1	1-hexanol	861	858	0.2 ± 0.02
2	ethylangelate	918	920	0.1 ± 0.00
3	*α*-pinene	935	942	35.8 ± 2.11
4	camphene	950	954	1.0 ± 0.03
5	*β*-myrcene	985	991	6.2 ± 0.03
6	limonene	1020	1023	27.6 ± 1.14
7	*α*-ocimene	1038	1042	4.1 ± 0.04
8	linalool	1071	1089	3.2 ± 0.03
9	fenchol	1091	1098	2.0 ± 0.05
10	*trans*-allocimene	1128	1125	0.1 ± 0.01
11	L-borneol	1160	1152	0.7 ± 0.06
12	terpinen-4-ol	1175	1170	0.1 ± 0.00
13	*α*-terpineol	1185	1190	1.1 ± 0.04
14	farnesane	1386	1381	0.1 ± 0.01
15	hexyl caproate	1390	1386	0.1 ± 0.00
16	*trans*-*α*-bergamotene	1433	1430	0.2 ± 0.02
17	*β*-caryophyllene	1439	1435	9.6 ± 0.11
18	α-santalene	1448	1449	0.2 ± 0.02
19	aromadendrene	1455	1451	0.3 ± 0.04
20	humulene	1457	1454	1.9 ± 0.04
21	*α*-farnesene	1462	1461	0.3 ± 0.02
22	*β*-bisabolene	1498	1495	1.3 ± 0.04
23	*trans*-*α*-bisabolene	1549	1545	0.7 ± 0.02
24	trans-nerolidol	1551	1547	0.2 ± 0.02
25	caryophyllene oxide	1615	1613	0.4 ± 0.02
26	guaiol	1622	1625	0.8 ± 0.03
27	*γ*-eudesmol	1627	1630	0.6 ± 0.02
28	*β*-eudesmol	1652	1649	0.3 ± 0.02
29	hexaydrofarnesylacetone	1851	1846	0.2 ± 0.02
30	*m*-camphorene	1966	1960	0.4 ± 0.02
31	*p*-camphorene	1998	1994	0.1 ± 0.01
	SUM			99.9
	Monoterpenes			82.0
	Sesquiterpenes			17.5
	Others			0.4

^1^ The components are reported according to their elution order on apolar column; ^2^ Linear retention indices measured on apolar column; ^3^ Linear retention indices from the literature.

**Table 2:** Chemical composition (percentage mean value ± standard deviation) of *C. sativa* L. Hy, as determined by LVI-GC/MS.

N°	COMPONENT ^1^	RI ^2^	Hy
1	4-hydroxy-butanoic acid	15.16	10.0 ± 0.12
2	hexanoic acid	16.18	3.4 ± 0.09
3	linalool	16.67	3.2 ± 0.09
4	fenchol	17.36	1.3 ± 0.07
5	α-terpineol	19.15	14.5 ± 0.13
6	linalool oxide	22.67	6.7 ± 0.10
7	limonene-1,2-diol	24.50	5.2 ± 0.09
8	8-hydroxylinalool	24.87	4.2 ± 0.12
9	sobrerol	25.34	6.4 ± 0.09
10	3-buten-1,2-diol-1(2-furanyl)-2-methyl	27.24	16.1 ± 0.08
11	sobrerol acetate	27.36	9.6 ± 0.08
12	2-methylisoborneol	29.06	19.2 ± 0.11
	SUM		99.8
	Monoterpenes		60.7
	Sesquiterpenes		-
	Others		29.5

^1^ The components are reported according to their elution order on apolar column; ^2^ Retention time.

**Table 3:** Chemical composition (percentage mean value ± standard deviation) of *C. sativa* L. Hy as determined by DI-SPME/GC-MS.

N°	COMPONENT ^1^	LRI ^2^	LRI ^3^	Hy
1	1-hexanol	852	858	2.8 ± 0.04
2	5-hpten-2-one	860	866	0.2 ± 0.02
3	ethyl dimethylacrylate	918	924	0.6 ± 0.03
4	*α*-pinene	937	942	0.7 ± 0.02
5	camphene	951	954	0.2 ± 0.02
6	*β*-myrcene	987	991	2.6 ± 0.04
7	1,8-cineole	1028	1031	3.0 ± 0.03
8	linalool oxide	1068	1073	0.3 ± 0.02
9	fenchone	1072	1080	2.2 ± 0.05
10	linalool	1091	1089	23.2 ± 0.15
11	fenchol	1097	1098	21.2 ± 0.18
12	L-borneol	1155	1152	6.8 ± 0.06
13	terpinen-4-ol	1167	1170	2.0 ± 0.02
14	*α*-terpineol	1193	1190	26.4 ± 0.21
15	*β*-caryophyllene	1430	1435	1.9 ± 0.03
16	humulene	1461	1454	0.4 ± 0.02
17	*β*-bisabolene	1499	1495	0.3 ± 0.02
18	epi-*γ*-eudesmol	1615	1610	0.6 ± 0.03
19	humulene epoxide II	1617	1611	1.2 ± 0.04
20	guaiol	1631	1625	1.5 ± 0.02
21	*γ*-eudesmol	1634	1630	1.3 ± 0.03
22	*β*-eudesmol	1648	1649	0.6 ± 0.02
	SUM			100.0
	Monoterpenes			88.6
	Sesquiterpenes			7.8
	Others			3.6

^1^ The components are reported according to their elution order on apolar column; ^2^ Linear retention indices measured on apolar column; ^3^ Linear retention indices from the literature.

**Table 4:** IC_50_ values of the tumor cells exposed to EO or Hy for 72 h.

Tumor Cell Line	IC_50_ EO (%)	IC_50_ Hy (%)
A375	0.065 ± 0.015	3.72 ± 1.10
LOX IMVI	0.081 ± 0.069	3.71 ± 0.67
H1299	0.088 ± 0.017	3.43 ± 0.88
A549	0.101 ± 0.089	4.311 ± 1.07
HT29	0.017 ± 0.001	7.702 ± 2.57
L3.6	0.017 ± 0.003	7.314 ± 1.08

## Data Availability

Data available on request due to restrictions of privacy. The data presented in this study are available on request from the corresponding author.
